# University of Maryland Dental Department

**Published:** 1889-06

**Authors:** 


### Editorial, Etc.

---

University of Maryland Dental Department, Drs. Wright's and Wardlaw's Statements Confirmed by the Secretary of "The Arkansas State Dental Examining Board."—Dr. M. C. Marshall, of Little Rock, Ark.; the Secretary of the Arkansas State Board of Dental Examiners, in a letter to a member of the class of 1888-89 of the University of Maryland Dental Department, dated March 3d, 1889, writes as follows: "The Arkansas State Board is a member of the National Board of Dental Examiners. The National Board at its last meeting (in Louisville) adopted a list of Colleges whose diplomas should be recognized by the different State Boards in lieu of an examination, and the Dental Department of the University of Maryland is *left out*. I was at that meeting and heard the merits and demerits of all other Colleges that were left out discussed, but heard nothing in regard to the Dental Department of the University of Maryland. To my mind this was an ominous silence in the light of subsequent events, viz: the publication of the list of Colleges left out, and the correspondence that followed between the Secretary of the National Board, Prof. Gorgas, Dr. Wright, of South Carolina, and others. It looks a little ugly; has the appearance of a 'sub rosa' act."

Dr. Marshall also says that the Arkansas State Board would register a dentist on the diploma of the University of Maryland Dental Department.

No State Dental Board has refused to accept the diploma of this dental school.

